# Antimicrobial Activity of Common Mouthwash Solutions on Multidrug-Resistance Bacterial Biofilms

**DOI:** 10.4021/jocmr1535w

**Published:** 2013-08-05

**Authors:** Majed M. Masadeh, Shadi F. Gharaibeh, Karem H. Alzoubi, Sayer I. Al-Azzam, Wasfi M. Obeidat

**Affiliations:** a Department of Pharmaceutical Technology, Faculty of Pharmacy, Jordan University of Science and Technology, Irbid 22110, Jordan; b Department of Clinical Pharmacy, Faculty of Pharmacy, Jordan University of Science and Technology, Irbid 22110, Jordan

**Keywords:** Mouthwash, Antimicrobial, Biofilm, Planktonic

## Abstract

**Background:**

Periodontal bacteria occur in both planktonic and biofilm forms. While poor oral hygiene leads to accumulation of bacteria, reducing these microbes is the first step toward good oral hygiene. This is usually achieved through the use of mouthwash solutions. However, the exact antibacterial activity of mouthwash solution, especially when bacteria form biofilms, is yet to be determined. In this study, we evaluated the antibacterial activity of common mouthwash solutions against standard bacteria in their planktonic and biofilm states.

**Methods:**

Standard bacterial strains were cultured, and biofilm were formrd. Thereafter, using standard method for determination of minimum inhibitory concentrations (MIC) values of various mouthwash solutions were determined.

**Results:**

Results show that common mouthwash solutions have variable antibacterial activity depending on their major active components. Only mouthwash solutions containing chlorohexidine gluconate or cetylpyridinum chloride exhibited activity against majority, but not all tested bacterial strains in their biofilm state. Additionally, bacteria are generally less susceptible to all mouthwash solutions in their biofilm as compared to planktonic state.

**Conclusions:**

While mouthwash solutions have variable antibacterial activity, bacteria in their biofilm state pose a challenge to dental hygiene/care where bacteria become not susceptible to majority of available mouthwash solutions.

## Introduction

When unicellular organisms come together forming a community and attached to a solid surface then encased in an exo-polysaccharide matrix, bacterial biofilms are formed [1-3]. These biofilms can be made up of single or multiple bacterial species. As periodontal biofilms are more resistance to antibiotics than planktonic cells, their control pauses a real problem, and requires special care of mouth hygiene [4, 5].

More than three hundred known species of bacteria are considered as oral flora [6]. They include species in both panktonic and biofilm states [1-2]. Poor oral hygiene causes accumulation of metabolites of these oral floras, through which dental caries, gingivitis, and periodontitis will be induced [7-9]. Reducing these microbes is the first step in the endodontic therapy as it enhances the normal healing process of the periodontal tissues [10].

Mouthwash solutions are aimed at ensuring sufficient oral hygiene. Its ease of use in addition to the significant ability to reduce dental plaque formation, made mouthwashes a reasonable method to limit gingivitis and periodontitis [4, 11-13]. However, the consumer faces a wide range of options of mouthwash solutions that differ in their deodorant, analgesic or astringent property and antimicrobial effectiveness [14-16]. This different effect on oral flora in addition to the differences in the susceptibility to antimicrobials in mouthwash solutions between biofilms and planktonic cells highlights the importance of screening oral antimicrobial formulations in an effort to produce more predictive of clinical activity [5, 15, 17]. In this study, we evaluated the effect of different mouthwash solutions on variety of bacteria both in their biofilm and planktonic states.

## Materials and Methods

### Mouthwash solutions

Mouthwash solutions were obtained from the local market. Table 1 shows the active ingredients, and pH of each of the mouthwash solutions tested.

**Table 1:** Active Ingredients and pH of Mouthwash Solutions

Mouthwash	Active ingredients	pH
A	Cetylpyridinium chloride 0.05% w/w, sodium fluoride 0.05% w/w	6.3
B	Sodium bicarbonate 2% w/v, sodium benzoate	8.4
C	Chlorhxidine gluconate 0.2% w/v	3.9
D	Potassium aluminum sulphate 0.1%, Chlorhexidine gluconate 0.1% w/v, ethanol (96%) 10%, Menthol crystals 0.045%	1.9
E	Chlorhexidine gluconate 0.2% w/v, ethanol 7.9%	5.7
F	Chlorhexidine gluconate 0.2% w/v	5.4
G	Hexetidine 0.1% w/v	3.1
H	Povidone -iodine 1% w/v	2.5
I	Eucalyptol 0.092%, Menthol 0.042%, Methylsalicylate 0.060%, thymol 0.064%	4.1

### Bacterial strains and media

The following bacterial strains were purchased and used in the study: *Escherichia coli* (ATCC 25922), *Enterococcus faecalis* (ATCC 19433), *Staphylococcus aureus* (ATCC 29213), *Methicillin Resistans Staphylococcus Aureus MRSA* (ATCC 43300), *Pseudomonas aeruginosa* (ATCC 27853), *Staphylococcus epidermidis* (ATCC 12228), *Klebsiella pneumoniae* (ATCC 13883), *Streptococcus pyogenes* (ATCC 19615), *Proteus mirabilis* (ATCC 12453), and *Acinitobacter baumanni* (ATCC 19606). All strains were stored in trypticase-soy broth with 20% glycerol (BBL Microbiology Systems, Cockeysville, Md., USA) at -70 °C in a deep freezer until ready for susceptibility testing. They were thawed and passed 3 times to assure purity and viability before each experiment.

### Biofilm formation, harvesting, and screening

Biofilm cells were performed as described by [18] under standardized aseptic conditions. Briefly, 100 µL of bacterial suspension from each strain was cultivated in polypropylene tubes containing 2 mL of Trypticase Soy Broth (TSB) supplemented with 1% glucose for 48 hours at 37 °C. Culture media was refreshed after 24 hours of incubation. After 48hr of incubation, biofilm cells were harvested by discarding the culture media and washing the tubes three times with phosphate buffer saline (PBS, pH 7.2), to remove non adherent bacteria, and then the adhered cells were harvested by vortex and centrifugation. The pellet was suspended in PBS (pH 7.2) to achieve the desired turbidity (comparable to a 0.5 McFarland turbidity standard). Screening for biofilms formation was achieved as previously described in [19]. Briefly, after being emptied from their content, culture tubes were stained with trypan blue or safranin. Biofilms were judged by the appearance of a visible film lined the walls of the tube. Observations were carried out by three independent observers. Biofilms were scored as absent (score 0), weak (score 1), moderate (score 2), or strong (score 3). The average scores were used.

### Determination of minimum inhibitory concentrations (MIC) values

The MIC values of both *S. aureus* and *P. aeruginosa* planktonic and biofilm cells were tested against selected antibiotics, MIC were determined by using broth macrodilution method according to NCCLS [20]. Briefly, 100 µL of adjusted bacterial suspensions equivalent to 0.5 McFarland standard were added to a 2-fold serial dilutions of selected antibiotics diluted in Mueller Hinton broth. The results were observed after 24 hours of incubation at 37 °C. The lowest concentration of antibiotic needed to inhibit microbial growth compared to control culture was defined as MIC. Tests were performed in triplicate for each antibiotic.

## Results

The MIC values of tested mouthwash solutions against bacterial planktonic strains are shown in Table 2. Mouthwash A has antimicrobial activity against all strains except *P. aeruginosa* and *K. pneumoniae*. Mouthwashes B, C, D, and E, have activity against all strains used in the study. In addition, mouthwash F exhibits high activity (low MIC values) against all strains except *K. pneumoniae*. However, mouthwashes H, G, and I show activity against some strains, but no activity against others. Although mouthwash B is active against all strains, its MIC values are larger than those for mouthwashes C, D, and E for almost all strains. Moreover, mouthwash B shows lower activity (larger MIC values) except for *K. pneumoniae* when compared with mouthwash F. This indicates that mouthwash B exhibits less activity than C, D, E, and F. Mouthwash I has some activity against all bacterial strains except *S. epidermidis*, *S. pyogenes*. Moreover, it has low activity (MIC values > 25%) against *MRSA, K. pneumoniae*, and *A. baumannii*. Mouthwash H exhibits little activity (no activity or high MIC values) against planktonic bacterial strains.

**Table 2:** Minimum Inhibitory Concentration (MIC) Values for Tested Products Against Bacterial Strains in Both Their Planktonic, and Biofilm Forms

Species	Product	A	B	C	D	E	F	G	H	I
*E. coli*	Planktonic	1.3 ± 0.46	33.3 ± 14.43	2.6 ± 0.92	20.9 ± 7.16	7.3 ± 4.67	5.6 ± 8.52	10.4 ± 3.58	50.0 ± 0.00	12.5 ± 0.00
	Biofilm	16.7 ± 7.16	50.0 ± 0.00	-	41.7 ± 14.43	50.0 ± 0.00	16.7 ± 7.22	14.6 ± 9.53	-	12.5 ± 0.00
*S. aureus*	Planktonic	0.7 ± 0.23	41.7 ± 14.40	16.7 ± 7.22	25.0 ± 0.00	1.3 ± 0.47	1.8 ± 1.22	2.3 ± 1.33	-	8.3 ± 3.61
	Biofilm	20.9 ± 7.16	33.3 ± 14.43	25.0 ± 0.00	25 ± 0.00	25.0 ± 0.00	25.0 ± 0.00	29.2 ± 19.09	-	50.0 ± 0.00
*E. faecalis*	Planktonic	16.7 ± 7.16	25.0 ± 0.00	0.6 ± 0.20	33.3 ± 14.43	2.6 ± 0.92	2.4 ± 1.39	2.6 ± 0.92	50.0 ± 0.00	12.5 ± 10.83
	Biofilm	10.5 ± 3.70	50.0 ± 0.00	29.2 ± 19.09	41.7 ± 14.43	41.7 ± 14.43	25.0 ± 0.00	33.3 ± 14.43	-	-
*P. aeruginosa*	Planktonic	-	33.3 ± 14.43	33.3 ± 14.43	33.3 ± 14.43	7.3 ± 4.76	8.3 ± 3.61	50.0 ± 0.00	50.0 ± 0.00	20.8 ± 7.22
	Biofilm	50.0 ± 0.00	-	41.7 ± 14.43	50.0 ± 0.00	18.8 ± 10.83	18.75 ± 10.8	50.0 ± 0.00	50.0 ± 0.00	33.3 ± 14.43
*MRSA*	Planktonic	6.2 ± 0.00	16.7 ± 7.22	1.9 ± 0.46	1.1 ± 0.46	3.2 ± 0.00	1.8 ± 1.22	1.1 ± 0.40	-	50.0 ± 0.00
	Biofilm	-	33.3 ± 14.43	7.3 ± 4.77	8.3 ± 3.70	16.7 ± 7.22	41.7 ± 14.43	16.7 ± 7.22	-	-
*S. epidermidis*	Planktonic	5.1 ± 1.85	20.8 ± 7.22	0.6 ± 0.20	16.7 ± 7.16	1.1 ± 0.46	1.6 ± 0.00	0.7 ± 0.23	16.7 ± 7.22	-
	Biofilm	6.2 ± 0.00	25.0 ± 0.00	29.2 ± 19.09	41.7 ± 14.43	25.0 ± 0.00	50.0 ± 000	29.1 ± 19.09	41.7 ± 14.43	-
*A. baumannii*	Planktonic	33.3 ± 14.43	25.0 ± 21.65	1.1 ± 0.46	2.5 ± 0.81	1.6 ± 0.00	3.9 ± 3.42	4.2 ± 1.85	-	25.0 ± 0.00
	Biofilm	41.7 ± 14.43	41.7 ± 14.43	41.7 ± 14.43	8.3 ± 3.70	50.0 ± 0.00	50.0 ± 0.00	41.7 ± 14.43	-	-
*P. mirabilis*	Planktonic	33.3 ± 14.43	33.3 ± 14.43	1.6 ± 1.39	25.0 ± 0.00	1.8 ± 1.22	1.8 ± 1.22	-	50.0 ± 0.00	41.7 ± 14.43
	Biofilm	25.0 ± 0.00	50.0 ± 0.00	-	41.7 ± 14.43	-	-	-	-	-
*S. pyogrnes*	Planktonic	1.9 ± 1.22	50.0 ± 0.00	10.4 ± 3.61	2.5 ± 0.81	41.7 ± 14.4	6.7 ± 7.63	2.9 ± 2.99	25.0 ± 0.00	-
	Biofilm	50.0 ± 0.00	-	33.3 ± 14.43	25.1 ± 21.59	33.3 ± 14.43	25.0 ± 21.65	27.1 ± 21.93	23.3 ± 2.89	-
*K. pneumoniae*	Planktonic	-	29.2 ± 19.09	2.1 ± 0.92	33.3 ± 14.43	12.5 ± 0.00	-	1.6 ± 1.33	50.0 ± 0.00	50.0 ± 0.00
	Biofilm	-	50.0 ± 0.00	-	25.0 ± 0.00	41.7 ± 14.43	-	50.0 ± 0.00	-	-

Values are presented as mean ± SD of 3 experiments.

Table 2 also lists MIC values for tested mouthwashes against bacterial biofilm strains. The results show that the MIC values of tested mouthwashes against biofilm strains are greater compared with the planktonic strains. Among the tested mouthwashes, A, C, D, E, and F exhibit MIC values for most of the tested bacterial strains. However, mouthwashes B, H, and I show either higher MIC values or no activity for almost all strains.

## Discussion

Mouthwash solutions usually encompass antimicrobial activity that ensures their work in eliminating harmful periodontal bacteria, which aids in preventing future dental carries, gingivitis and periodontitis [4, 11-13]. In here, we showed that mouthwash solutions are generally less effective against bacteria in their biofilm compared to planktonic forms. Additionally, common mouthwash solutions were shown to possess variable antibacterial activity against bacteria in their biofilm state depending on their major active components.

Bacterial biofilms formation is associated with poor dental hygiene that provides better environment for biofilms formation, thus, rendering oral bacteria less susceptible to mouthwash or similar antiseptic formulation [5, 15, 17]. Therefore, keeping good oral hygiene, which is expected to help in avoiding the biofilms formation, is recommended to ensure maintaining susceptibility of oral bacteria to mouthwash and other dental hygiene procedures.

Results of this study show that mouthwash solution possesses variable antibacterial activity depending on their chemical composition. For example, mouthwash solutions containing the antiseptics chlorhexidine are effective on most oral bacterial strains. This correlates with previous studies and is related to chlorohexidine’s mode of action as it works on different sites of the bacteria [21, 22]. Additionally, mouthwash solutions containing sodium bicarbonate 2% were shown to be effective against most of the tested bacterial strains, which is in agreement of previous studies [23, 24].

On the other hand, mouthwash solutions containing other ingredients such as cetylpyridinium chloride, sodium fluoride, hexetidine, povidone-iodine, eucalyptol, menthol, methylsalicylate, and thymol showed activity against some bacterial strains, but not others. For example, cetylpyridinium chloride (0.05%) mouthwash was shown to possess antimicrobial activity against all tested bacterial strains except *P. aeruginosa* and *K. pneumoniae*. In fact, cetylpyridinum chloride is a quaternary ammonium compound known for its use as a cationic surface active agent that has antibacterial activity [15, 25, 26]. Moreover, the MIC values for cetylpyridinium chloride (0.05%) mouthwash suggest that it has high activity against *S. aureus*. This is in agreement with previously published work on cetylpyridinium chloride products [27].

The eucalyptol mouthwash has some activity against all bacterial strains except *S. epidermidis, S. pyogenes*. Moreover, It has low activity (MIC values > 25%) against *MRSA, K. pneumoniae*, and *A. baumannii*. This mouthwash contains active ingredients of eucalyptol, menthol, methylsalicylate, and thymol. Eucalyptol, which is present in the essential oil of Eucalyptus, has been reported to posses some antibacterial activity against *S. aureus* and *E.coli* [28-30]. However, it has limited activity against other bacterial strains [28-30]. In addition, menthol and thymol were reported to have limited antibacterial activity [31].

The hexitidine 0.1% mouthwash has high activity against all strains except *P. mirabilis*. Upon comparison to mouthwashes that contains chlorhexidine as the main active ingredient, hexetidine 0.1% mouthwash exhibited less antibacterial activity against almost all strains. This is in agreement with work reported by Aznita et al [32].

Results of the current study show that the MIC values of tested products against biofilm strains are greater compared with the planktonic strains. This is in agreement with what is known about biofilm resistance to anti microbial agents [4, 5]. Several mechanisms for increased antimicrobial resistance of the biofilms have been suggested [1, 2, 5]. It is also reported that multiple resistance mechanisms can be working in a single biofilm community [33]. Among the tested mouthwash solutions, containing chlorohexidine gluconate or cetylpyridinum chloride exhibited certain antibacterial activity against majority of tested biofilm bacterial strains. However, other mouthwashes limited or no antibacterial activity for most strains. This is in agreement with previous studies showing that cetylpyridinum chloride and chlorohexidine gluconate have activity against bacterial strains in their biofilm state [34, 35].

Collectively, it is been shown that bacteria in their biofilm state is less susceptible to mouth wash solutions. Only mouthwash solutions containing chlorohexidine gluconate or cetylpyridinum chloride exhibited activity against majority, but not all tested bacterial strains in their biofilm state.
